# Efficacy of Fat Supplements with Different Unsaturated/Saturated FA Ratios Undergoing First Postpartum Ovulation in Lactating Anovulatory Goats

**DOI:** 10.3390/vetsci12010060

**Published:** 2025-01-15

**Authors:** Caroline P. Silva, César C. L. Fernandes, Juliana P. M. Alves, Camila M. Cavalcanti, Felipe B. B. Oliveira, Alfredo J. H. Conde, Diana Celia S. N. Pinheiro, Darcio I. A. Teixeira, Anibal C. Rego, Davide Rondina

**Affiliations:** 1 School of Veterinary Medicine, Ceará State University (UECE), Fortaleza 60714-903, CE, Brazil; caroline.silva@ifap.edu.br (C.P.S.); juli.alves@uece.br (J.P.M.A.); camila.muniz@uece.br (C.M.C.); alfredo.herrera@aluno.uece.br (A.J.H.C.); diana.pinheiro@uece.br (D.C.S.N.P.); darcio.teixeira@uece.br (D.I.A.T.); 2 Health Sciences Center, University of Fortaleza (UNIFOR), Fortaleza 60811-905, CE, Brazil; caancesar@gmail.com; 3 Institute of Animal Health and Production, Amazônia Federal Rural University (UFRA), Belém 66077-830, PA, Brazil; felipe.brener@yahoo.com.br; 4 Animal Science Department, Federal University of Ceará, Fortaleza 60455-760, CE, Brazil; anibalcr@ufc.br

**Keywords:** goat, first ovulation, microalgae, linseed, dietary supplementation, fat

## Abstract

We investigated whether microalgae or linseed supply during the early postpartum period affects ovarian restimulation and supports the first postpartum ovulation in lactating anovulatory goats. Thirty-eight An-glo-Nubian-crossbred adult goats were allocated into three groups, one with a control diet (*n* = 12), fed a total mixed ration (TMR) comprising chopped elephant grass and concentrate; an algal diet (*n* = 13), fed TMR + green microalgae (1% dry matter); and a linseed diet (*n* = 13), TMR + linseed (12% dry matter). Supplements were furnished from the second to fifth week (time of weaning). Goats were estrus synchronized on day 40 by insertion of an intravaginal CIDR device for 5 days, after which 0.075 mg PGF2α was applied to induce ovulation, and estrus was monitored for 72 h. From the 5th–15th day of ovulation induction, the corpus luteum (CL) area and progesterone rate were monitored. The algal and linseed groups showed lower feed intake (*p* < 0.001) and higher (*p* < 0.001) triglyceride levels/follicle numbers, respectively. After estrus induction, no differences were observed in estrus response; however, the linseed group showed more and larger growing follicles (*p* = 0.016 and *p* < 0.01), a higher ovulation rate (*p* < 0.05), a larger CL area (*p* < 0.05), and higher progesterone levels (*p* < 0.001). Linseed after delivery stimulates follicular growth before and after ovulation induction, favoring better CL quality during the first ovulation.

## Simple Summary

In the postpartum period, pregnancy losses during the first mating still represent the main obstacle to reproductive expression in ruminants. The first ovulation and gestational success are dependent on the animal’s ability to recover from the effects of negative energy balance and in turn depend on the choice of an appropriate nutritional strategy. In goats, as in other ruminants, recent attention has been given to the use of green microalgae and flaxseed as dietary supplements. These products have high concentrations of polyunsaturated fatty acids and exert their effects at low doses in the diet. Therefore, the objective of this study was to verify the efficacy of these two supplements in anovulatory goats in a challenging environment from a reproductive point of view, such as the early postpartum period. The results showed that flaxseed successfully stimulated follicular growth, ovulatory response, and corpus luteum quality in the animals; on the contrary, this did not occur with green microalgae. Furthermore, the latter, also used in low doses, reduced food consumption, which casts doubt on the sustainability of its use over longer periods.

## 1. Introduction

In ruminants, the first ovulation and the presence of a functional corpus luteum in the postpartum period are indispensable to ensure pregnancy after insemination [1], as well as the production of quality oocytes [2] in in vitro embryo culture. In dairy cows, postpartum ovarian function is strictly associated with metabolic conditions before and after calving, which in turn are associated with the appearance of pro-inflammatory pathologies in periparturient periods [1]. Accentuated lipolysis, due to a negative energy balance, promotes oxidative stress that damages DNA, proteins, and lipids, thus favoring immune system dysfunction [3]. Oxidative stress also increases the pro-inflammatory phenotype of immune cells, which act on redox-regulated pro-inflammatory factors [4]. In addition, changes in the molecular profile also occur due to the upregulation of pro-inflammatory genes, such as the cytokines IL-1β and TNF-α [5].

Data on pregnancy losses at the first service in goats are limited, largely because of the conditions of management in an extensive system [6]. In tropical and subtropical regions, the main goat breeders and high environmental temperatures make the postpartum reproductive management of herds even more difficult due to the difficulty in meeting nutritional demands with adequate food consumption [7]. Consequently, nutritional strategies play a fundamental role in this field, and the use of lipid food sources is widely recognized, owing to their high energy content [8], their ability to optimize rumen fermentations with lower methane production [9], and the impact of polyunsaturated fatty acids (PUFAs) on the reproductive response [10].

In one study in goats, [11] achieved a resumption of upper follicular activity on day 5 postpartum and a reduction in serum prostaglandin F2α on day 7 postpartum through diet supplementation with PUFA-rich fish oil (−3 to +3 weeks postpartum). The metabolism of prostaglandins can be influenced by the dietary intake of n-6:n-3 PUFAs, which are directly associated with embryonic survival both in vivo and in vitro [12]. In addition to the positive effects of n-3 PUFA supplementation on different reproductive processes such as follicular turnover and growth, the development of the corpus luteum and steroidogenesis modulate ovarian dynamics [12,13].

Despite these results, the primary challenge in this field is to quantify the lipid requirements in reproductive processes, such as breeding time [14]. High levels of fat in ruminant feed (>5%) have limitations due to the negative effects on feed utilization and digestibility [8]. Recent studies in goats have further shown that higher fat inclusion (7%) for short periods (11 days) stimulates follicular growth without harming the animals [15]; however, modulating lipid supplementation to promote reproductive response at longer intervals, such as postpartum and in the presence of high metabolic stress, remains a challenge. Recently, several authors have focused on products, such as green microalgae [16,17,18] and flaxseed [19,20], both of which have a high content of unsaturated fatty acids and the advantage of being used in smaller quantities in the diet, allowing for control of the baseline dry matter fat. Although their use is promising, inconsistencies remain regarding the effectiveness of long-term supplementation [19,21], as well as the actual efficacy of un-saturated fatty acids on oocyte quality and embryonic development [22,23].

Thus, we hypothesized that the inclusion of green microalgae or flaxseed in diets for lactating goats during the postpartum period would effectively promote follicular growth and sustain the conditions for the first ovulation. Therefore, the present study aimed to investigate the impact of green microalgae (*Chlorella pyrenoidosa*) and flaxseed supplementation during the first month postpartum in the diets of lactating goats on follicular growth, oxidative and metabolic stress, and ovarian and luteal responses before and after ovulation induction using a hormonal synchronization protocol.

## 2. Materials and Methods

### 2.1. Animals and Diet Management

This study was conducted at the School of Veterinary Medicine farm, Ceará State University (UECE), Brazil. All procedures in this study were approved by the Ethics Committee on Animal Experimentation of UECE (no. 38257569). The model animals included 38 Anglo-Nubian crossbred adult and pluriparous goats with 33.6 ± 6.6 kg body weight, a 2.7 ± 0.2 (score from 1 to 5) body condition score, and an age of 42.0 ± 8.6 months (overall mean ± SD). All animals belonging to the school farm herd had their estrus synchronized and were naturally mated with Anglo-Nubian males. Pregnancies were detected using ultrasonography 45 days after mating, when they were clinically followed up by a trained veterinarian.

All goats received the same diet, comprising a total mixed ration (TMR) of chopped elephant grass and concentrate. The TMR was prepared in a water solution and furnished to satisfy the nutritional requirements of adult non-dairy goats [24] for each phase of gestation (initial and late) and early lactation. The TMR was prepared immediately before feeding and offered twice, at 08:00 and 15:00. Food refusals were collected and weighed daily to estimate diet intake.

### 2.2. Nutritional Treatments and Experimental Design

**Figure 1:**
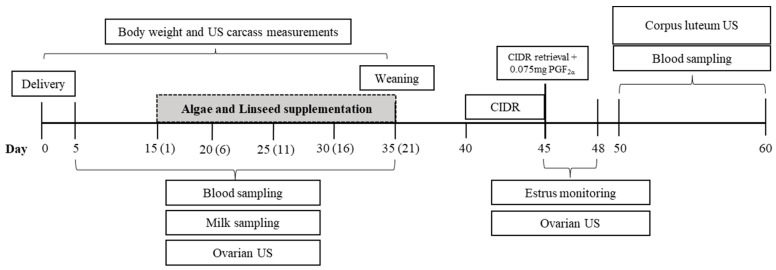
Timeline for the experimental design used in the study, describing the period of dietary supplementation and the hormonal protocol for estrus synchronization.

At delivery, goats were distributed homogeneously according to the feeding group as follows: control diet (*n* = 12) fed with a baseline TMR, algal diet (*n* = 13), and linseed diet (*n* = 13). In the latter two groups, the TMR was supplemented with 1% DM of powdered green microalgae (*Chlorella pyrenoidosa*) or ground linseed on a 12% DM basis, respectively. Supplements were added to the TMR concentrate and offered from the 2nd week to the 5th week after delivery (Figure 1). Goats were distributed to maintain a similar ratio between pregnancy types (six singletons and seven twins in the Algae and Linseed groups; five singletons and seven twins in the control diet) and were kept in open collective shed stalls, grouped according to feeding and pregnancy type, with free access to mineral supplements and water. After delivery, the kids were kept with goats and weaned at the 5th week postpartum.

The foodstuff ingredients and chemical compositions of the diets and supplements are presented in Table 1. For microalgae, lipids were quantified gravimetrically according to [25]. To evaluate the acid profiles, samples were analyzed using a gas–liquid chromatograph coupled to a mass spectrometer (model GCMS-QP2010S, SHIMADZU SCIENTIFIC INSTRUMENTS, Kyoto, Japan). Non-fibrous carbohydrate (NFC) values were calculated as described by [26]. The metabolizable energy (ME) diet content was calculated according to the following recommendations in [24]: ME (Mcal/kg of DM) = TDN (kg) × 4.4 × 0.82.

### 2.3. Synchronization Program

At 40 days postpartum, five days after the weaning of kids (Figure 1), all goats underwent synchronization of the estrus and follicular wave through the insertion of an intravaginal device impregnated with progesterone (CIDR^®^, controlled intravaginal drug release; InterAg, Hamilton, New Zealand). After five days, the device was removed, and 1 mL (0.075 mg) of prostaglandin (PGF2α) (Prolise^®^; ARSA S.R.L., Buenos Aires, Argen-tina) was applied to induce ovulation. Following CIDR removal, estrus was monitored by a trained observer for 72 h using a vasectomized buck introduced into the pen four times daily (06:00, 10:00, 14:00, and 18:00).

### 2.4. In Vivo Performance and Adipose Carcass Marker Measurements

Every five days, from delivery to the 5th week postpartum (Figure 1), goats were weighed and adipose mass was verified by ultrasonography by measuring the thickness of the subcutaneous lumbar fat deposits [27]. To estimate the levels of visceral fat, we measured the thickness of the kidney fat behind the 13th rib using a previously described methodology [28]. A convex transducer with a frequency of 3.5 MHz (Z5 Vet; Mindray Bio-Medical Electronics Co., Shenzhen, China) was used for kidney imaging. Images were captured in triplicate and analyzed using the previously calibrated ImageJ program (ImageJ, National Institutes of Health, Bethesda, MD, USA).

### 2.5. Ovarian Function

#### Follicular Dynamics, Ultrasonography Analysis, and Ovulatory Rate

Ultrasonography was performed daily from the 5th to the 35th day after delivery and during the 72 h after prostaglandin administration, corresponding to ovulation induction. Ovarian images were obtained with B-mode ultrasound equipment using a 5 MHz linear transrectal probe (model Z5 Vet; Mindray Bio-Medical Electronics Co., Shenzhen, China). Images were captured and analyzed using Image J software (Version 1.54g, National Institutes of Health, Millersville, MA, USA), which was previously calibrated.

An ovarian follicular wave was defined as the emergence of a group of small follicles (<3 mm) that gave rise to one or more large follicles (≥3 mm). The day of wave emergence was defined as the day when the largest follicle of the wave reached 3 mm in diameter. The growth phase was defined as the period during which a large follicle grows from 3 mm to its maximum diameter. The regression phase was defined as the period from the maximum follicle diameter to a diameter of 3 mm. Six days after the injection of the PGF2 alfa analog (Figure 1), the ovulatory response was quantified by laparoscopy through a morphological classification of the corpus luteum, as described previously [29].

### 2.6. Luteal Function

#### 2.6.1. Progesterone Assay

Blood samples were collected every five days from the fifth day after parturition until weaning and every two days from the fifth to fifteenth day after ovulation induction (Figure 1). Samples were taken by jugular venipuncture into heparinized vacuum tubes (BD Vacutainer^®^, Franklin Lakes, NJ, USA), performed before the morning feed was provided. Samples were then centrifuged at 3000 rpm for 15 min to separate the plasma, from which three aliquots were obtained and stored at −20 °C until progesterone analysis. Progesterone analyses was performed on an automatic analyzer (IMMULITE^®^ 2000, Siemens Healthcare Diagnostics LTDA, Llanberis, UK) using a specific commercial kit (IMMULITE^®^ 2000 progesterone, Siemens Healthcare Diagnostics LTDA, Llanberis, UK), with a sensitivity of 1 ng/mL, as indicated by the manufacturer’s recommendations.

#### 2.6.2. Postpartum Luteal Activity

Luteal activity was monitored from the fifth day after parturition until weaning by measuring the plasma progesterone concentrations, which indicated the presence of corpora lutea. The corpus luteum was considered functional when progesterone concentrations were greater than 1 ng/mL in at least two successive samplings, non-functional when progesterone concentrations were greater than 1 ng/mL in less than two consecutive samplings, and absent when progesterone concentrations were less than 1 ng/mL.

#### 2.6.3. Corpus Luteum Ultrasonography

Luteal activity after ovulation induction was measured daily using B-mode ultrasonography from the fifth day after ovulation induction (Figure 1). Evaluations were performed using B-mode ultrasound equipped with a 5.0 MHz linear transrectal probe for the identification and counting of the corpus luteum (CL), as described previously [30]. In brief, B-mode and Doppler-mode videos of both ovaries were recorded for subsequent evaluation. In the B-mode, frozen images were measured for CL count and luteal diameter (mm). Images corresponding to the cross-section of the CL at its greatest diameter were collected to obtain the total ovarian area [30].

#### 2.6.4. Metabolites, β-Hydroxybutyrate (BHB), Glutathione Peroxidase (GPx) Assays, and Fat–Protein Milk Ratio

Blood samples were collected every five days from the fifth day after parturition until weaning and at the fifth day after ovulation induction using heparinized vacutainer tubes (BD Vacutainer^®^, Franklin Lakes, NJ, USA) before morning feeding. The samples were subsequently centrifuged at 3000 rpm for 15 min to separate the plasma, from which three aliquots were obtained and stored at −20 °C for further quantification of metabolites. Plasma concentrations of cholesterol and triglycerides were determined using an automated biochemical analyzer (Mindray BS 120, Mindray^®^) and commercial kits (Bioclin, Quibasa, Minas Gerais, Brazil). The sensitivity of the assay kit was 1.472 mg/dL and 2.58 mg/dL for cholesterol and triglycerides. GPx and BHB were analyzed using an automated biochemical analyzer (Mindray BS 120, Mindray^®^) and commercial kits (Randox Laboratories, Crumlin, UK) with a sensitivity of 75U/L for GPx and 0.100 mmol/L for BHB.

Every five days from the fifth day after parturition until weaning, milk samples were taken and analyzed for fat and protein concentrations (Lactoscan SA^®^, Entelbra, Londrina, Brazil). The fat-to-protein ratio (FPR) of milk was calculated as the milk fat content (g/kg) divided by the milk protein content (g/kg).

### 2.7. Statistical Analysis

Statistical analyses were performed using Statistica Software version 13.4.0.14 (2018; TIBCO Software, Inc., Palo Alto, CA, USA). All data were initially verified for mathematical assumptions using the Shapiro–Wilk test. If these conditions were not met, log10x transformation was applied. All descriptive data were analyzed using the GLM procedure analysis of variance (ANOVA). The factors used in the model included diet (Control, Algae, Linseed), the interval between samples used (time), and interactions. The type of parturition (single or twin) effect and diet × type of parturition interaction were tested for measurements before the hormonal induction of ovulation and were not significant (*p* > 0.05) for most of the parameters; thus, only the main effects of diet and time were presented. All pairwise comparisons were performed using the Newman–Keuls post hoc test. The number of CL classes, number of does in estrus, and number of does that ovulated were compared using the chi-squared test.

## 3. Results

### 3.1. Response During Supplementation

#### 3.1.1. Changes in Feed Intake, Body Weight, and Carcass Markers

**Figure 2:**
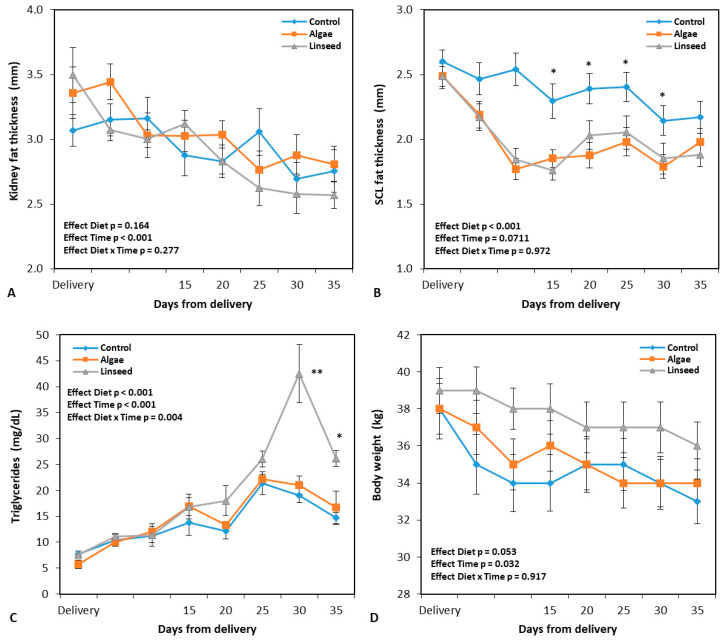
Kidney fat thickness (**A**), subcutaneous loin fat thickness (**B**), triglyceride levels (**C**), and body weight (**D**), with measurements performed after delivery and during nutritional supplementation from 2 weeks to 5 weeks after delivery (21 days) in goats. Data are plotted as mean ± SEM. The *p*-value for the ANOVA effects for supplementation period are shown in the figure. Time, ANOVA effect for interval of assessment used. * *p* < 0.05, ** *p* < 0.01, differences between groups.

The group supplemented with microalgae showed a reduced (*p* < 0.001) dry matter intake (Table 2) compared to the control group and animals supplemented with linseed. When intake was expressed as a percentage of body weight, the microalgae intake was 2.9%, whereas in the other treatments, it reached 3.4%. Figure 2 illustrates the changes after delivery for body weight and carcass adipose tissue marker levels after delivery. For the measurements performed during the supplementation interval, no differences were observed between the nutritional groups in terms of body weight (*p* = 0.053) (Figure 2D). All three groups lost an average of 1.5 kg of mass, which was equivalent to 4.2% of their own weight. If the total interval between delivery and weaning of the kids was considered (Figure 2D), the average losses were 4.6 kg, equivalent to 12% of the initial weight of the females.

All animals showed a reduction in kidney fat thickness (time effect: *p* < 0.001; Figure 2A). This reduction was on average 0.3 mm for the supplementation interval and 0.6 mm between delivery and weaning, respectively. Regarding lumbar subcutaneous fat (Figure 2B), both supplemented groups recorded a smaller thickness than the control (*p* < 0.001) during dietary treatment. None of the groups showed any significant effect on the measurement interval (time effect, *p* = 0.0711). Figure 2B presents how the most consistent reduction in adipose deposits occurred between delivery and the beginning of supplementation.

#### 3.1.2. Metabolic and Oxidative Damage Markers

No differences were recorded between the groups regarding the plasma concentrations of cholesterol and Gpx (Table 2). An increase in cholesterol levels was observed between days 6 and 11 of supplementation (effect time: *p* < 0.001; Table 2). Regarding plasma β-hydroxybutyrate, no differences were also evidenced between the groups (Table 2).

The plasma concentrations of triglycerides are presented in Figure 2C. Overall, our analysis revealed a significant interaction between diet and time (*p* = 0.004), owing to the substantial increase in metabolites in the animals supplemented with linseed from the second week of supplementation (Figure 2C), which did not occur in the other groups that showed a decrease in triglyceride levels in the same interval. The milk fat/protein ratio (Table 2) was higher in the supplemented groups than in the control group.

#### 3.1.3. Ovarian and Luteal Function

**Figure 3:**
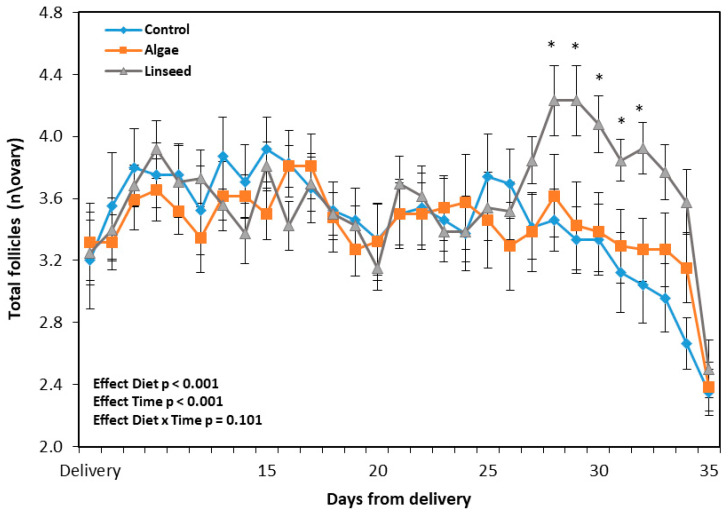
Total follicles counted by ultrasonography performed after delivery and during nutritional supplementation from 2 weeks to 5 weeks after delivery (21 days) in goats. Data are plotted as mean ± SEM. The *p*-value for the ANOVA effects for the supplementation period are shown in the figure. Time, ANOVA effect for interval of assessment used. * *p* < 0.05 differences between groups.

Figure 3 presents the number of follicles counted using ultrasound analysis after delivery and supplementation. There was a diet effect (*p* < 0.001) owing to a significant increase in the ovarian follicular population in the linseed group between the 14th day and the 18th day of supplementation (Figure 3).

The presence of corpora lutea was verified using peripheral progesterone concentrations (Table 2). During the supplementation period, no functional corpora lutea were observed, and one non-functional CL was recorded in the linseed group (Table 2). This result justified the higher progesterone levels (*p* < 0.05; Table 2).

### 3.2. Response After Ovulation Induction

#### 3.2.1. Responsiveness to Estrus Synchronization

The groups did not differ in terms of their responses to hormonal treatment for estrus synchronization (Table 3). However, in the control group, we observed a significant difference between the number of goats in estrus and the total number of synchronized animals (Table 3). The overall mean number of animals in estrus was 29% (11/38), with the onset of estrus at 34 h following CIDR withdrawal. Although the supplemented groups recorded an estrus duration twice that of the control group, the difference was not statistically significant (Table 3). Thus, the average estrus duration was 28 h.

#### 3.2.2. Follicles, Ovulatory Response, BHB, and GPx

During the measurement interval, all groups showed a significant increase (Table 3) in the number of large follicles (time effect, *p* = 0.009) and the maximum follicular diameter (time effect, *p* = 0.006). However, a comparison of the groups revealed that animals supplemented with linseed showed higher values for both parameters (Table 3). At the same time, in the same nutritional treatment, a significant reduction in the number of small follicles (<3 mm) was also recorded. There were no differences in the number of ovulating does between the diets (Table 3); however, in the control group, a significant difference (*p* < 0.05) was observed between the number of ovulated animals and the total number of synchronized batches. Regarding the ovulation rate (Table 3), the supplemented groups showed values twice as high as those of the control, with a significant difference observed between the linseed and control group (*p* < 0.05).

No differences were observed between nutritional treatments in relation to the concentrations of β-hydroxybutyrate in glutathione peroxidase (Table 3).

#### 3.2.3. Luteal Function

**Figure 4:**
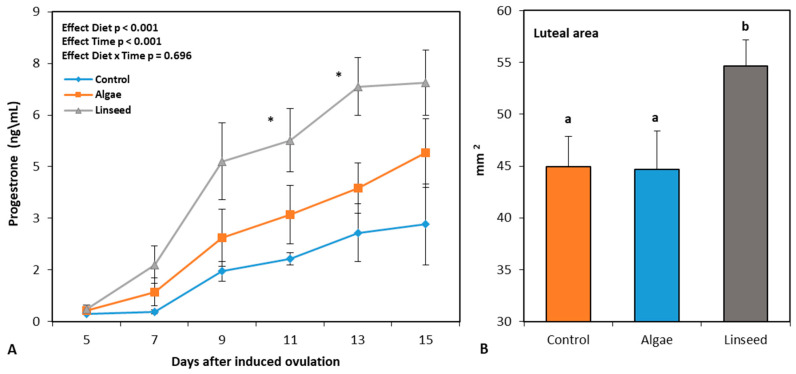
Peripheral progesterone levels (**A**) and luteal area (**B**), measured after ovulation induction by hormonal treatment started 40 days after delivery in goats previously supplemented with algae or linseed. Data are plotted as mean ± SEM. (**A**) The *p*-value for the ANOVA effects of diet group; the interval of assessments and interaction are shown in the figure. a,b *p* < 0.05 differences between nutritional groups, shown in (**B**). * *p* < 0.05, differences between groups.

Progesterone concentrations in the CL measured after estrus induction revealed that only one CL was classified as non-functional in the linseed group. Figure 4 presents the progesterone levels recorded following ovulation induction in the functional CL (Figure 4A) and luteal areas (Figure 4B). The linseed group had a larger luteal area than the control and microalgal groups. All three nutritional treatments showed an increase in peripheral progesterone during the measurement interval (time effect, *p* < 0.001; Figure 4A); however, the linseed group had higher values starting from day 11 after ovulation induction.

## 4. Discussion

Overall, the results of the present study partially confirm our initial hypothesis, showing that the inclusion of linseed in the diet between the second and fifth weeks postpartum stimulated ovarian and luteal responses in goats. The data also showed that among the two sources of lipids chosen, only flaxseed was successful, whereas the use of microalgae, at least at the proposed dosages, did not present advantages in terms of re-productive responses.

These two supplements have specific nutritional characteristics and different inclusion intervals in ruminant diets. However, in the case of microalgae, the results were not as promising as expected. This is surprising, as prior studies have shown it is possible to obtain a significant follicular growth response using low dosages of microalgae [16,17,18], which allows for maintenance of the baseline dry matter fat of the diet for ruminants between 2 and 3%.

Microalgae are a potential source of lipid supplementation due to their ability to produce biomass rich in polyunsaturated fatty acids (PUFAs), such as ω-3 eicosapentaenoic acid (EPA; 20:5 n-3) and docosahexaenoic acid (DHA; 22:6 n-3) [31]. Chlorella sp. is a species of green microalgae that has been widely used as a supplement in the diet of ruminants, not only because of the beneficial action of PUFAs on milk quality [32,33] but also to improve reproductive parameters, such as ovarian response [16] and embryo production [18]. This supplement at the cell membrane level affects the transfer of nutrients to reproductive tissues [34], as well as precursors of steroids and prostaglandins. Reference [18] observed that the administration of 10 g of Chlorella in a short time interval (7 days) induced a lower diet consumption but simultaneously promoted greater intraovarian blood perfusion and an increase in the number of large follicles. This phenomenon can be explained by the higher plasma glucose levels promoted by the greater availability of AF [35], since glucose is an intrafollicular mediator that, at higher concentrations in the blood, promotes an increase in insulin-like growth factor 1 (IGF1) and inhibits the action of neuropeptide Y, which acts on the metabolic hormonal axis and stimulates the release of GnRH in the hypothalamus, which in turn increases FSH and LH pulses, thereby stimulating follicular growth [36].

Conversely, research has shown that the supplementation of diets with products rich in PUFAs for a longer period can promote a decrease in feed intake due to a reduction in the palatability and digestibility of fiber, which can alter the pH and biohydrogenation during rumen fermentation, causing toxic effects in the rumen [32]. This phenomenon may have been responsible for the lower use of the microalgal diet and, ultimately, for the change in the milk protein/fat ratio in the two supplemented groups. The use of high doses of microalgae, for example, leads to substantial changes in the use and digestibility of feed; however, a reduction in dry matter intake has also been recorded with brief supplementation [17,18], without having an impact on the ovarian response.

In our study, flaxseed supplementation induced an increase in plasma triglycerides between days 25 and 30 postpartum, which corresponded to the third week of diet application. In the same time interval, an increase in the ovarian follicular population was also observed through ultrasound analysis. The flaxseed diet also resulted in more efficient follicular growth following hormonal synchronization. Ultrasound analysis revealed more intense follicular depletion (a reduction in the number of small follicles) in this group, in parallel with a greater increase in the number of large follicles and follicular size. This phenomenon did not occur at the same intensity in the other treatments.

As previously anticipated, the lack of efficacy of the microalgal diet was not an expected result; the literature shows that the dosage used in shorter application times is sufficient to induce an increase in lipid metabolites [37] and to stimulate follicular dynamics [18]. The discrepancy between these results is potentially attributable to the low nutritional status of the animals in the present study as a consequence of prolonged metabolic imbalance in the postpartum period. At the supplementation interval, all experimental groups showed renal adipose mass mobilization, with an average weight loss of 130 g/day, values that were in accordance with those reported in [15,38]. In addition, the results showed that the concentrations of peripheral GPx, an indicator of oxidative stress, and BHB remained high in all groups at the measurement intervals.

After calving, energy needs are met through adipose tissue catabolism, an adaptation mechanism that increases the plasma levels of ketone bodies such as β-hydroxybutyric acid and non-esterified fatty acids. In turn, the increase in lipolysis is strictly associated with oxidative stress and the establishment of inflammatory states in immune cells, with the consequent production of cytokines that act on pro-inflammatory factors regulated by redox reactions. During this phase, cytokines diffuse in a paracrine/autocrine manner to regulate ovarian function and are involved in the control of follicular development and ovulation, acting on follicular development, steroidogenesis, ovulation, corpus luteum function, and the regulation and differentiation of granulosa cells [39]. TNF-α is a mediator of ovulation in terms of oocyte release and granulosa cell autophagy in ovarian tissue remodeling and favoring luteinizing hormone action has shown that luteinizing hormone (LH) induces ovulation via TNF-α-dependent increases [40,41]. As such, the BEN nadir is temporarily associated with a marked increase in LH pulse rate, which facilitates the first postpartum ovulation [1].

The two supplements also behaved differently with respect to ovulation and functionality of the corpus luteum induced by the synchronization protocol. Although both groups expressed a low response to the synchronization protocol in terms of animals in estrus, the flaxseed diet displayed a higher rate of ovulation and luteal area and, consequently, a higher secretion of progesterone during the development of the CL. The latter two parameters are usually used as indicators of CL functionality and quality [30], which in turn represent key factors for fertilization and embryonic development during breeding [30].

In this context, flaxseed supplementation promoted a positive effect on the ovarian response. The inclusion level used in the diet was allowed to remain within the fat level limit range indicated for ruminants [8] and simultaneously provided a higher energy density in the diet. Thus, it is plausible that the difference in the flaxseed diet in terms of ovarian response was an effect of the availability of an extra share of food energy and the greater presence of n-3 fatty acids, which are rich in flaxseed. Flaxseed has been used in ruminant supplementation because it contains approximately 40–45% PUFAs in its composition, mainly alpha-linolenic, a precursor of EPA and DHA [42], which modulates the activation of the primordial follicle and stimulates folliculogenesis by promoting the increase in IGF1 levels [43]. In addition, IGF1 induces the expression of P450 cholesterol desmolase and aromatase, both of which are enzymes essential for the production of cholesterol, a precursor of progesterone, directly influencing ovarian metabolic processes [44,45,46]. In goats, supplementation with flaxseed at 8–12% DM promoted an increase in the levels of fatty acids in milk [47,48,49] and quality embryos [50], whereas in cows, it resulted in the accumulation of linolenic acid and its metabolites in milk and plasma [37], increased follicular growth and oocyte quality, and reduced pregnancy losses [42,51].

However, there are contrasting results regarding the effect of PUFA supplementation on oocyte chemical properties, embryo quality, and fertility, as the composition of follicular fluid depends on the reproductive age of the animal, follicular size, and type of PUFAs administered [2,23]. According to [23], the follicular fluid of adult and prepubertal goats, percentages of linolenic acid (ALA; n-3 PUFA), total saturated fatty acids (SFAs), and total n-3 PUFAs were significantly higher in adults, and the percentages of linoleic acid (LA; n-6 PUFA), total MUFAs, total PUFAs, total n-6 PUFAs, and n-6: n-3 PUFAs were higher in prepubertal goats than in adults. However, in prepubertal goats, comparing large (≥3 mm) and small (<3 mm) follicles, the concentrations of total SFAs, MUFAs, PUFAs, and n-6 PUFAs were higher in the small ones. In in vitro studies, the concentrations of linolenic and linoleic acids added as supplements to the IVM medium affected the competence of oocytes for embryo development [52]. Other studies have indicated that pregnancy rates increase in cattle-fed diets containing high amounts of n-3 fatty acids [53]. In addition, n-3-enriched diets may inhibit PGF2α synthesis, preventing the regression of the corpus luteum [54], and as a result, there are sustained concentrations of progesterone required for embryo survival and pregnancy maintenance.

## 5. Conclusions

The addition of linseed to the diet in the first few weeks following parturition in goats induced effective follicular growth from the second week of supplementation, and the synchronization protocol was applied at 40 days postpartum. Furthermore, the use of this food allowed for the expression of efficient ovarian activity and better quality of the corpus luteum in hormonally induced ovulation. From these results, we concluded that under the experimental conditions of the present study, dietary supplementation with linseed is an efficient strategy to support the first ovulation in goats.

## Figures and Tables

**Table 1:** Ingredients and chemical composition of diets in goats fed with algae or linseed from 2 weeks to 5 weeks after delivery.

Parameters	Diet			Algae	Linseed
	Control	Algae	Linseed		
Ingredients, g/kg DM					
Elephant grass	600	600	600	-	-
Ground corn grain	120	120	60	-	-
Soybean meal	120	120	100	-	-
Wheat bran	140	140	100	-	-
Ground linseed	-	-	120	-	-
Algae	-	10	-	-	-
Mineral mixture	20	20	20	-	-
Chemical fraction					
Dry matter, g/kg as-fed basis	568	523	573	892	929
Crude protein, g/kg of DM	114	102	122	397	245
Ether extract, g/kg of DM	28	28	57	210	283
Neutral detergent fiber, g/kg of DM	546	507	555	178	331
Acid detergent fiber, g/kg of DM	310	297	329	50	231
Ash, g/kg of DM	89	83	88	44	34
Non-fibrous carbohydrates, g/kg of DM	324	430	420	917	910
ME, Mcal/kg of DM	2.23	2.27	2.35	-	-
Fatty acids, %					
Saturated	-	-	-	28.1	11.5
Unsaturated	-	-	-	71.9	88.5
Monounsaturated	-	-	-	45.0	23.1
Polyunsaturated	-	-	-	26.9	65.4
UFA/SFA *				2.6	7.7

* UFA: unsaturated fatty acid; SFA: saturated fatty acid.

**Table 2:** Dry matter intake, glutathione peroxidase, BHB and luteal function recorded in goats from 2 weeks to 5 weeks after delivery, during the 21 days of nutritional supplementation with algae or linseed.

Parameters	Diet				*p*-Value		
	Control	Algae	Linseed	SEM	Diet	Time	D vs. T
Feed intake							
DMI *, kg/doe	1.2 ^a^	1.0 ^b^	1.2 ^a^	0.015	<0.001	0.231	0.997
Metabolic markers and oxidative stress							
Glutathione peroxidase, U/L	127.3	120.5	147.8	2.169	0.058	0.947	0.702
BHB **, mmol/L	0.3	0.3	0.4	0.019	0.878	-	-
Cholesterol, mg/dL	53.6	63.2	62.9	1.774	0.107	<0.001	0.180
FPR ***, ratio	0.8 ^a^	1.0 ^b^	1.1 ^b^	0.03	0.002	0.822	0.994
Luteal function							
Functional CL ****, n/doe	0 ^B^	0 B	0 ^B^	-	-	-	-
Non-functional CL, n/doe	0 ^B^	0 B	1 ^B^	-	-	-	-
CL absent, n/doe	12 ^A^	13 ^A^	12 ^A^	-	-	-	-
Progesterone, ng/mL	0.2 ^a^	0.2 ^a^	0.3 ^b^	0.011	0.025	0.945	0.988

* DMI: dry matter intake; ** BHB: β-hydroxybutyrate, measured at beginning of supplementation; *** fat–protein milk ratio; **** CL: corpus luteum; Time, ANOVA effect for interval of assessment used. ^a,b^ *p* < 0.05 differences between diets. ^A,B^ *p* < 0.05 differences between CL classes in each diet.

**Table 3:** Reproductive responses, glutathione peroxidase, and BHB recorded after ovulation induction by hormonal treatment started 40 days after delivery in goats previously supplemented with algae or linseed.

Parameters	Diet				*p*-Value		
	Control	Algae	Linseed	SEM	Diet	Time	D vs. T
Follicle after ovulation induction *							
Follicles < 3 mm, n\ovary	2.0 ^ab^	2.0 ^a^	1.6 ^b^	0.032	0.044	0.094	0.503
Follicles ≥ 3 mm, n\ovary	1.4 ^ab^	1.2 ^a^	1.6 ^b^	0.025	0.016	0.009	0.404
Total follicles, n\ovary	3.3	3.2	3.3	0.025	0.461	0.681	0.503
Max follicle size, mm	4.2 ^a^	4.6 ^ab^	5.0 ^b^	0.051	0.008	0.006	0.175
Estrus and ovulatory response							
No. of does in estrus, % (n\n)	25.0 (3/12) ^‡^	38.5 (5/13)	46.2 (6/13)	-	0.367	-	-
Onset estrus **, h	38.6	41.7	21.5	6.278	0.344	-	-
Estrus length, h	16.3	31.5	37.7	6.036	0.473	-	-
No. of does ovulated, % (n\n)	25.0 (3/12) ^‡^	69.2 (9/13)	61.5 (8/13)	-	0.216	-	-
Ovulatory rate, n\doe	0.4 ^a^	0.8 ^ab^	1.0 ^b^	0.132	0.046	-	-
Metabolic marker and oxidative stress ***							
Glutathione peroxidase, U/L	188.9	235.6	228.6	2.211	0.345	-	-
BHB ****, mmol/L	0.2	0.2	0.3	0.038	0.715	-	-

* Follicle traits, measured by ultrasonography in the 72 h after ovulation induction; ** interval between CIDR removal and estrus onset; *** performed at 5th day after prostaglandin injection; **** BHB: β-hydroxybutyrate; Time, ANOVA effect for interval of assessment used. ^a,b^ *p* < 0.05 differences between diets; ^‡^ *p* < 0.05, chi-squared test in each diet.

## Data Availability

The data presented in this study are available upon request from the corresponding author due to legal reasons.
